# Supplementary material for: Effects of Action Observation Plus Motor Imagery Administered by Immersive Virtual Reality on Hand Dexterity in Healthy Subjects
Source: Bioengineering (Basel). 2024 Apr 19;11(4):398. doi: 10.3390/bioengineering11040398 (PMC11048356; doi:10.3390/bioengineering11040398)
Supplement: Supplementary file 1 [file bioengineering-11-00398-s001.zip › bioengineering-2874776-supplementary.pdf]

Table S1. Comparison between VR-AOMI, AOMI and Control groups at T0, T1 and T2 for kinematic indexes during Nine-Hole Peg Test performed with the left hand. Data are shown as mean and standard deviation. Significant results are shown in bold text.

	**VR-AOMI**			**AOMI**			**CTRL**					
	**T0**	**T1**	**T2**	**T0**	**T1**	**T2**	**T0**	**T1**	**T2**	**Time factor**	**Group factor**	**Time x Group interaction**
Test Total Time (s)	22.93 ± 2.76	22.96 ± 3.47	22.45 ± 3.38	22.95 ± 3.51	22.42 ± 3.82	22.68 ± 3.78	22.73 ± 3.92	23.16 ± 4.3	21.58 ± 4.01	0.077	0.973	0.560
Removing Tot Time (s)	6.97 ± 0.95	6.95 ± 1.38	6.65 ± 1.24	6.85 ± 1.13	6.66 ± 1.34	5.91 ± 1.7*	7.08 ± 1.28	7.08 ± 1.31	6.37 ± 1.09	**0.003**	0.535	0.484
Peg Grip Tot Time (s)	3.94 ± 0.87	4.09 ± 1.09	4.41 ± 1.30	4.17 ± 1.46	4.11 ± 1.20	4.60 ± 1.71	4.09 ± 1.20	4.08 ± 1.23	4.08 ± 1.26	0.055	0.882	0.349
Transfer Tot Time (s)	4.18 ± 0.67	4.00 ± 0.56	3.8 ± 0.65*	3.86 ± 0.38	3.73 ± 0.55	3.81 ± 0.50	3.88 ± 0.92	3.87 ± 0.81	3.75 ± 0.77	**0.040**	0.637	0.284
Peg in Hole Tot Time (s)	3.75 ± 0.70	3.66 ± 0.77	3.64 ± 0.62	4.08 ± 1.03	3.89 ± 1.09	3.60 ± 0.64	3.31 ± 0.67	3.84 ± 0.82	3.50 ± 0.83	0.261	0.475	0.088
Return Tot Time (s)	2.87 ± 0.38	2.92 ± 0.40	2.77 ± 0.27	2.77 ± 0.38	2.84 ± 0.39	2.65 ± 0.28	2.98 ± 0.50	3.06 ± 0.64	2.75 ± 0.43**	**0.029**	0.368	0.696
N-Jerk Transfer (m/s^3)	272.38 ± 79.43	255.35 ± 60.22	231.39 ± 62.43	236.99 ± 40.98	219.82 ± 53.25	234.75 ± 45.04	240.03 ± 84.51	232.76 ± 75.95	228.59 ± 69.19	0.058	0.499	0.221
N-Jerk Return (m/s^3)	170.36 ± 41.78	174.69 ± 44.25	157.53 ± 25.12	163.83 ± 53.90	163.73 ± 42.93	148.22 ± 30.65	186.68 ± 60.76	197.41 ± 72.99	160.35 ± 45.07	0.051	0.214	0.815
Vel Transfer (m/s)	0.27 ± 0.29	0.26 ± 0.29	0.27 ± 0.18	0.26 ± 0.2	0.26 ± 0.27	0.27 ± 0.01	0.27 ± 0.03	0.27 ± 0.04	0.28 ± 0.03	**0.042**	0.419	0.437
Vel Return (m/s)	0.32 ± 0.05	0.31 ± 0.04	0.32 ± 0.03	0.31 ± 0.05	0.29 ± 0.02	0.33 ± 0.03	0.32 ± 0.03	0.31 ± 0.03	0.34 ± 0.03	0.062	0.679	0.306
Peak Vel Transfer (m/s)	0.58 ± 0.09	0.54 ± 0.08	0.57 ± 0.07	0.56 ± 0.07	0.56 ± 0.11	0.57 ± 0.07	0.58 ± 0.11	0.58 ± 0.09	0.61 ± 0.09	0.274	0.642	0.320
Peak Vel Return (m/s)	0.72 ± 0.14	0.66 ± 0.09	0.72 ± 0.11	0.72 ± 0.11	0.68 ± 0.09	0.79 ± 0.11**	0.73 ± 0.09	0.69 ± 0.09	0.79 ± 0.13**	**0.009**	0.436	0.205

**p* <0.05 compared to T0 in the same group; ***p* <0.05 compared to T1 in the same group

Abbreviations: VR-AOMI: action observation performed through immersive virtual reality group; **AOMI**: action observation group; **CTRL**: control group.

Table S2. Comparison between VR-AOMI, AOMI and Control groups at T0 and T2 for kinematic indexes during Nine-Hole Peg Test (NHPT) performed with right hand. Data are shown as mean and standard deviation. Significant results are shown in bold text.

	**VR-AOMI**		**AOMI**		**CTRL**				
	**T0**	**T2**	**T0**	**T2**	**T0**	**T2**	**Time factor**	**Group factor**	**Time x Group interaction**
Test Total Time (s)	21.62 ± 2.84	20.51 ± 3.17	20.98 ± 2.71	22.05 ± 3.39	20.89 ± 3.06	19.91 ± 3.72	0.341	0.580	**0.025**
Removing Tot Time (s)	6.69 ± 1.54	6.23 ± 0.99*	6.71 ± 1.39	6.51 ± 0.97	6.50 ± 1.20	6.04 ± 1.15*	**0.003**	0.731	0.579
Peg Grip Tot Time (s)	3.82 ± 0.90	3.70 ± 1.19	3.66 ± 0.84	4.49 ± 1.75	3.74 ± 0.95	3.76 ± 1.21	0.178	0.613	0.076
Transfer Tot Time (s)	3.63 ± 0.59	3.48 ± 0.69	3.55 ± 0.50	3.78 ± 0.51	3.54 ± 0.51	3.39 ± 0.54	0.755	0.543	0.089
Peg in Hole Tot Time (s)	3.31 ± 0.51	3.13 ± 0.92	3.09 ± 0.74	3.43 ± 0.74	2.92 ± 0.42	2.67 ± 0.68**	0.780	0.078	**0.043**
Return Tot Time (s)	2.90 ± 0.46	2.78 ± 0.35	2.97 ± 0.45	2.78 ± 0.52	2.85 ± 0.51	2.90 ± 0.59	0.194	0.965	0.328
N-Jerk Transfer (m/s^3)	218.21 ± 59.90	210.34 ± 64.62	208.99 ± 45.02	233.22 ± 46.60	206.81 ± 49.81	197.41 ± 40.99	0.778	0.494	0.179
N-Jerk Return (m/s^3)	184.83 ± 49.99	171.61 ± 33.40	186.93 ± 58.09	165.83 ± 63.15	172.68 ± 53.36	183.51 ± 54.65	0.325	0.992	0.237
Vel Transfer (m/s)	0.28 ± 0.03	0.28 ± 0.04	0.28 ± 0.02	0.27 ± 0.01	0.28 ± 0.03	0.29 ± 0.02	0.517	0.705	0.103
Vel Return (m/s)	0.31 ± 0.05	0.33 ± 0.04	0.32 ± 0.03	0.32 ± 0.04	0.32 ± 0.04	0.32 ± 0.04	0.510	0.992	0.426
Peak Vel Transfer (m/s)	0.59 ± 0.09	0.60 ± 0.12	0.60 ± 0.09	0.57 ± 0.06	0.61 ± 0.09	0.60 ± 0.07	0.645	0.841	0.376
Peak Vel Return (m/s)	0.75 ± 0.12	0.77 ± 0.14	0.77 ± 0.09	0.76 ± 0.11	0.74 ± 0.12	0.78 ± 0.10	0.390	0.990	0.550

**p* <0.05 compared to T0 of the same group; ***p* <0.05 compared to AOMI group at the same Time point

Abbreviations: VR-AOMI: action observation performed through immersive virtual reality group; **AOMI**: action observation group; **CTRL**: control group.
